# Post-transcriptional regulation dominates protein biosynthesis in *Landoltia punctata* under biogas slurry stress

**DOI:** 10.3389/fpls.2025.1694864

**Published:** 2025-11-20

**Authors:** Xin-Li Gong, Jun-Yi Li, Jia-Zhou Li, Cheng Ran, Le-Le Zhou, Tian Zhou, Huan-Huan Su, Ting-Ting Lu, Shi-Lin Zhang

**Affiliations:** 1 Ecological Breeding Department, Guangdong Laboratory for Lingnan Modern Agriculture, Heyuan, China; 2 Institute of Animal Science Guangdong Academy of Agricultural Sciences, State key Laboratory of Livestock and Poultry Breeding, Guangzhou, Guangdong, China

**Keywords:** biogas slurry, integrative multi-omics analysis, *Landoltia punctata*, post-transcriptional regulation, protein production

## Abstract

**Introduction:**

Duckweed represents a promising alternative protein source, yet enhancing its protein content remains essential for large-scale applications. This study investigated how high nitrogen and phosphorus stress from biogas slurry affects protein accumulation in Landoltia punctata and explored the underlying molecular regulatory mechanisms.

**Methods:**

*L. punctata* were cultivated in 1/5 strength Hoagland medium supplemented with 0-5% pig farm biogas slurry. The experimental groups showing the highest (4%) and lowest (0%) protein content were selected for integrated transcriptomic and proteomic analyses. Differentially expressed genes (DEGs) and proteins (DEPs) were identified and functionally characterized.

**Results:**

Biogas slurry treatments significantly increased crude protein content in a concentration-dependent manner, with the 4% treatment showing the highest value of 24.18% compared to 18.13% in controls. Multi-omics analysis revealed a low correlation between mRNA and protein expression (R=0.1387), indicating dominant post-transcriptional regulation. Ribosomal proteins were significantly upregulated at the protein level without corresponding transcriptional changes, suggesting enhanced translation efficiency. Concurrently, key enzymes in amino acid catabolism were downregulated, potentially conserving substrates for protein synthesis. The photosynthetic system showed coordinated downregulation at both transcriptional and protein levels, with suppression of light-responsive genes and carbon fixation pathway components, indicating redirected carbon and energy flows toward nitrogen assimilation.

**Discussion:**

Biogas slurry enhances duckweed protein accumulation primarily through post-transcriptional regulation. Enhanced translation efficiency coupled with metabolic reallocation from photosynthesis to nitrogen assimilation optimizes protein synthesis. This first multi-omics perspective on post-transcriptional regulation under biogas slurry stress provides theoretical support for molecular breeding of high-protein duckweed.

## 1 Introduction

Food security remains an increasingly pressing global challenge, with global food demand projected to rise by 35% to 56% between 2010 and 2050 (van Dijk et al., 2021). In response, the development of alternative protein sources has emerged as a critical strategy for mitigating potential crises. Duckweed, a small aquatic floating plant, offers dual benefits of environmental remediation and resource production. It demonstrates high efficiency in removing nitrogen and phosphorus pollutants (removal rate >90%) and in adsorbing heavy metals from eutrophic water bodies (Liu et al., 2020; Rai and Nongtri, 2024). Additionally, its dry biomass contains 20-35% protein, comparable to soybean, and is rich in essential amino acids, positioning it as a sustainable candidate for alternative feed protein. Current research on duckweed has shown promising applications in both food and feed sectors. Appenroth et al. conducted a comprehensive nutritional analysis of all 11 species in the genus *Wolffia*, demonstrating their potential as potential human food sources (Appenroth et al., 2018); Gutiérrez et al. demonstrated that substituting sorghum and soybean meal with *Lemna gibba* in pig feed did not compromise production performance (Gutiérrez et al., 2001); Stadtlander et al. reported that feeding rainbow trout fry with *Spirodela polyrhiza* cultivated in liquid manure significantly enhanced growth performance (Stadtlander et al., 2019); and Shrivastav et al. found that replacing 10% of soybean meal with *S. polyrhiza* improved growth, digestive enzyme activity, and nutritional composition in carp (Shrivastav et al., 2022). Despite some duckweed species exhibiting protein levels comparable to soybeans, their protein content is often unstable and highly sensitive to environmental conditions. In most cases, it remains lower than that of soybeans. Therefore, identifying effective strategies to enhance and stabilize the protein content of duckweed is essential for supporting its broader adoption in the food and feed industries.

Previous studies have demonstrated that nutrient regulation, such as increasing the concentration of nitrogen and phosphorus in the growth medium, or environmental interventions like adjusting light intensity can effectively enhance protein accumulation in duckweed (Petersen et al., 2021, 2022). Notably, the successful application of genetic transformation techniques to stimulate and strengthen starch synthesis in duckweed has opened new avenues for boosting its protein content. Concurrently, biogas slurry, a nutrient-rich wastewater byproduct of livestock and poultry farming, contains high levels of nitrogen, phosphorus, and various heavy metals. If discharged directly, it poses a significant risk of causing eutrophication in aquatic ecosystems (Ke et al., 2022). While biogas slurry is primarily used in agriculture for crop irrigation, it remains unclear whether duckweed can simultaneously purify this wastewater and generate high-protein biomass in a coordinated and sustainable manner. Moreover, the molecular regulatory mechanisms underlying this potential dual function have yet to be systematically elucidated.

Transcriptomics and proteomics are essential technical tools for identifying key molecules involved in complex biological processes, and they have been extensively applied to characterize gene and protein expression patterns in duckweed under various treatments. Wang et al. reported that starch synthesis genes such as APS1, APL3, and GBSSI were significantly upregulated during abscisic acid (ABA)-induced dormancy in *S. polyrhiza* (Wang et al., 2014); Shang et al. analyzed the transcriptional response of *S. polyrhiza* to heat stress and identified key gene expression patterns associated with thermal tolerance (Shang et al., 2023); Zhang et al. found that treatment with sodium nitroprusside significantly promoted starch accumulation and antioxidant capacity in *S. polyrhiza* by activating flavonoid and nitric oxide (NO) synthesis pathways while suppressing photosynthetic gene expression (Zhang et al., 2024b); Yang et al. revealed differences in cadmium stress tolerance mechanisms between two duckweed species through transcriptome analysis, showing that the more tolerant strain responded by enhancing antioxidant activity and ribosome biosynthesis (Yang et al., 2023); Su et al. investigated *Lemna minor* under aluminum stress using proteomics and clarified its antioxidant defense mechanisms at the protein level (Su et al., 2019). In the case of *Landoltia punctata*, the species studied here, Tao et al. employed transcriptomic analysis to elucidate the molecular network associated with starch accumulation under nutrient-deficient conditions (Tao et al., 2013), while Huang et al. further demonstrated, through proteomic studies, the synergistic regulatory mechanism involving the activation of starch synthase and the inhibition of starch-degrading enzymes (Huang et al., 2014). Additionally, their findings revealed that uniconazole significantly enhances starch yield by modulating hormone and starch metabolism pathways (Huang et al., 2015).

Compared to single-omics approaches, integrated multi-omics analyses can overcome the limitations of studying individual molecular layers by systematically revealing the regulatory networks underlying complex biological processes through the correlation of gene, protein, and metabolite data. For instance, Muthan et al. used lipidomic and transcriptomic analyses to investigate lipid and carbohydrate responses to heavy metal stress (Muthan et al., 2024), while Shi et al. applied metabolomics, transcriptomics, and ^13^C-based flux analysis to explore *L. gibba*’s metabolic adaptation to different nitrogen sources, such as nitrate and glutamine (Shi et al., 2023). However, integrative transcriptomic–proteomic studies in duckweed are still scarce. A key exception is Fu et al., who, through combined transcriptome and proteome analysis, highlighted extensive post-transcriptional regulation during salicylic acid-induced flowering in *L. gibba*, underscoring its critical role in floral development (Fu et al., 2024).

In this study, *L. punctata* was cultivated in Hoagland medium supplemented with 0–5% pig farm biogas slurry. Samples from the 0% and 4% groups, representing the lowest and highest protein contents—were selected for transcriptomic and proteomic analyses. We identified differentially expressed genes (DEGs) and differentially expressed proteins (DEPs) associated with the increase in duckweed protein content induced by biogas slurry and examined the roles of these DEGs and DEPs when regulated either jointly or independently at the mRNA and protein levels. This research represents the first systematic investigation of the regulatory mechanisms governing protein synthesis in duckweed under biogas slurry stress using a multi-omics approach, offering novel insights into the post-transcriptional regulation of duckweed and laying the groundwork for future molecular and applied studies.

## 2 Materials and methods

### 2.1 Plant material and processing

*L. punctata* used in this study was obtained from the Heyuan Branch Center of the Lingnan Modern Agricultural Science and Technology Guangdong Provincial Laboratory (Guangdong Province, China). The biogas slurry was sourced from Dongrui Food Group Co., Ltd., with a total nitrogen (TN) content of 980 mg/L and a total phosphorus (TP) content of 90 mg/L. Cultivation was conducted in 1500 mL white plastic pots, with a control group consisting of 1000 mL of Hoagland nutrient solution (0% biogas slurry) and five treatment groups containing 1.5%, 2%, 3%, 4%, and 5% biogas slurry, corresponding to 15, 20, 30, 40, and 50 mL of slurry, respectively, supplemented with Hoagland solution to a final volume of 1000 mL. Plants were grown under controlled environmental conditions with a 16-hour light/8-hour dark photoperiod, a light intensity of 6000 LUX, and a constant temperature of 25 ± 1°C. Each treatment group was set up with three biological replicates. After 7 days of cultivation, 1.5 g of fresh plant material was collected, flash frozen in liquid nitrogen, and stored at −80°C for transcriptomic RNA-seq and proteomic analyses. The remaining biomass was used to determine the crude protein content.

### 2.2 Determination of crude protein content

Crude protein content was determined following the method outlined in the national standard GB/T 6432-2018. Samples were first subjected to overnight digestion with concentrated sulfuric acid, followed by further digestion using a KDN-20D digestion oven (Gedana, China) at 420°C for 10 minutes, until the solution became a clear blue color. The digested sample was then distilled using an N310 semi-automatic Kjeldahl nitrogen analyzer (Gerhardt, China) and titrated with 0.5 mol/L H_2_SO_4_ until the gray-red endpoint was observed. Ammonium sulfate was used as the nitrogen standard for quantitative calibration.

### 2.3 RNA-seq library construction and sequencing

The RNA-seq experiment was conducted by Guangzhou Gene Denovo Biotechnology Co., Ltd. (Guangzhou, China). Total RNA integrity was verified using 1% agarose gel electrophoresis, while RNA concentration and purity were assessed with a Nanodrop ND-2000 spectrophotometer (Thermo Scientific, USA) and an Agilent 2100 Bioanalyzer (Agilent, USA). Library preparation was performed using the Hieff NGS^®^ Ultima Dual-mode mRNA Library Prep Kit (Yeasen Biotechnology, Shanghai, China). Specifically, poly(A)+ mRNA was isolated and fragmented to construct a double-stranded cDNA library, which was then subjected to 150 bp paired-end sequencing on the Illumina NovaSeq X Plus platform. Raw sequencing reads were assembled using Trinity software, generating contiguous sequences (Unigenes) based on overlapping reads, containing no ambiguous nucleotides (N). Assembly quality was evaluated by analyzing N50 values, sequence length distribution, and Benchmarking Universal Single-Copy Orthologs (BUSCO) completeness (http://busco.ezlab.org/). Functional annotation of the Unigenes was performed using BLASTx alignment (E-value < 0.0001) against major protein databases, including NR, SwissProt, Kyoto Encyclopedia of Genes and Genomes (KEGG), and Clusters of Orthologous Groups (COG/KOG), with the highest-scoring protein match used to assign annotation information to each Unigene.

### 2.4 Identification of DEGs

Gene expression levels were normalized using FPKM (Fragments Per Kilobase of transcript per Million mapped reads) values. Expression quantification was performed using RSEM (Pertea et al., 2015), and Differentially Expressed Genes (DEGs) were identified using the edgeR (Robinson et al., 2010). DEGs were screened based on the criteria of |log_2_(fold change) | > 1 and a false discovery rate (FDR) < 0.05. To determine functional significance, DEGs were mapped to the Gene Ontology (GO) database (http://www.geneontology.org/) and the KEGG database (https://www.kegg.jp/). Subsequently hypergeometric enrichment tests were then performed to identify significantly overrepresented GO terms and KEGG pathways among the DEGs.

### 2.5 Protein preparation and LC-MS/MS analysis

The same batch of duckweed samples used for transcriptomic analysis was also employed for proteomic analysis. Total protein was extracted using the Plant Protein Extraction Kit (CWBIO, China), and protein concentration was quantified using the Bicinchoninic Acid (BCA) assay. Sample preparation, including protein denaturation, reduction and alkylation, trypsin digestion, and peptide desalting, was carried out using the iST sample pretreatment kit (PreOmics, Germany). The resulting peptide fractions were freeze-dried and stored at −80 °C. Prior to analysis, peptides were reconstituted and subjected to LC-MS/MS using a timsTOF Pro2 mass spectrometer (Bruker Daltonics, Germany) coupled with an UltiMate 3000 nano-flow liquid chromatography system (Thermo Fisher Scientific, USA). A total of 200 ng of each peptide sample was loaded onto an AUR3-15075C18 analytical column (15 cm × 75 μm i.d., 1.7 μm particle size, 120 Å pore size; IonOpticks). Peptide separation was achieved using a 60-minute gradient at a column temperature of 50 °C and a flow rate of 400 mL/min. The gradient profile started at 4% B phase (80% acetonitrile with 0.1% formic acid), increased to 28% over 25 minutes, then to 44% in the next 10 minutes, followed by an increase to 90% in another 10 minutes, held for 7 minutes, and finally returned to 4% for 8 minutes to re-equilibrate the column. Mass spectrometry data acquisition was performed in diaPASEF mode, with a scan range of 349–1229 m/z and a 40 Da isolation window. During PASEF MS/MS acquisition, collision energy was ramped linearly from 59 eV (1.6 Vs/cm²) to 20 eV (0.6 Vs/cm²) based on ion mobility (1/K_0_).

### 2.6 Identification of DEPs

Raw mass spectrometry data were processed using Spectronaut 18 (Bruker Daltonics) with parameters set to the BGS Factory Settings (default). The qualitative identification thresholds were defined as follows: a precursor FDR of 1.0% and a protein FDR of 1.0%. Protein quantification was performed using the MaxLFQ algorithm, applied to peptides with an FDR below 1.0%. All identified proteins were annotated using the GO, KEGG, and COG/KOG databases to obtain comprehensive functional information. Differentially Expressed Proteins (DEPs) were screened based on a |FC| > 1.5 and a Q-value < 0.05, corrected using the Benjamini-Hochberg method to control for multiple testing. DEPs were then mapped to the GO (http://www.geneontology.org/) and KEGG (https://www.kegg.jp/) databases, and hypergeometric tests were performed to identify significantly enriched GO terms and KEGG pathways.

### 2.7 Integration analysis of transcriptome and proteome

To correlate transcriptomic and proteomic data, KEGG annotation information was used to link genes and proteins assigned to the same KEGG Functional Ortholog (KO) number, thereby defining them as matched gene-protein pairs. DEGs and DEPs identified in the respective comparative groups were integrated and analyzed using a nine-quadrant plot classification. This analysis categorized expression patterns into groups such as Down-Down (simultaneously downregulated in both transcriptome and proteome), Up-Up (simultaneously upregulated), Up-Down (upregulated in transcriptome but downregulated in proteome), and Down-Up (downregulated in transcriptome but upregulated in proteome). Subsequently, based on all proteins identified in the proteomic dataset, functional enrichment analyses of the Down-Down and Up-Up gene/protein sets were conducted using GO and KEGG databases. These analyses aimed to compare functional preferences and identify common or distinct metabolic pathways between transcriptomic and proteomic responses. Additionally, Pearson correlation coefficients were calculated to assess the correlation between the expression levels of DEPs and their corresponding mRNAs.

### 2.8 Quantitative real-time PCR verification

Total RNA of duckweed was extracted using the Eastep^®^ Super Total RNA Kit (Promega, China). The extracted RNA was reverse-transcribed into cDNA using the EasyScript^®^ One-Step gDNA Removal and cDNA Synthesis SuperMix (TransGen, China). A total of 16 DEGs, including seven upregulated and nine downregulated genes, were selected for validation. The actin gene was used as the internal reference (**Supplementary Table S1**). qRT-PCR amplification was conducted using SYBR Green qPCR Master Mix (Vazyme, China) on the qTOWER3G real-time PCR system (Analytic Jena, Germany). The thermal cycling conditions were as follows: initial denaturation at 95°C for 30 s, followed by 40 cycles of 95°C for 10 s and 60°C for 30 s. Relative gene expression levels were calculated using the 2-ΔΔCT method (Livak and Schmittgen, 2001).

## 3 Results

### 3.1 Changes in protein content of *L. punctata* cultured in biogas slurry

**Figure 1:**
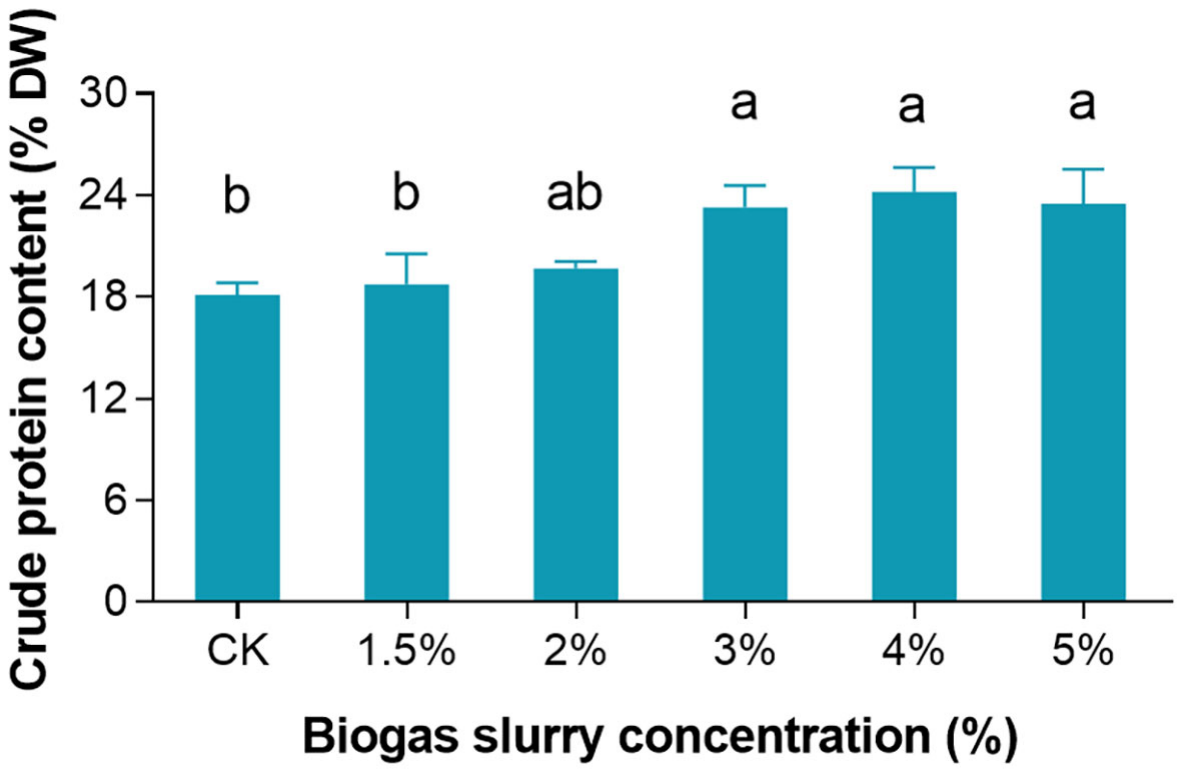
Crude protein content (% of dry weight, DW) of *Landoltia punctata* cultured for 7 days in biogas slurry at different dilution ratios. Data are presented as mean ± standard deviation of three biological replicates. Different letters above bars indicate significant differences according to Tukey’s honestly significant difference (HSD) test (P < 0.05).

To evaluate the effect of biogas slurry on the protein content of *L. punctata*, this study employed Hoagland’s nutrient solution without biogas slurry (0%) as the control group and established five treatment groups with biogas slurry concentrations of 1.5%, 2%, 3%, 4%, and 5%. After seven days of cultivation, crude protein content was measured using the Kjeldahl nitrogen determination method. The results showed that biogas slurry treatments led to varying degrees of protein content increase in *L. punctata*. The protein content in the control group was 18.13%, whereas the treatment groups exhibited values of 18.75%, 19.70%, 23.26%, 24.18%, and 23.50% for the 1.5%, 2%, 3%, 4%, and 5% concentrations, respectively. Statistical analysis revealed that the crude protein content in the 3% and 5% treatment groups was significantly higher than in the control group (P < 0.05), whereas the 4% treatment group showed a highly significant increase compared to the control (P < 0.01) (**Figure 1**).

### 3.2 RNA-seq analysis of *L. punctata*

Single-stranded RNA sequencing (ssRNA-seq) was performed on six libraries using the Illumina NovaSeq X Plus platform, comprising three biological replicates for the blank group (LpCK-1, LpCK-2, LpCK-3) and three for the treatment group (LpTreat-1, LpTreat-2, LpTreat-3). After quality filtering, the numbers of clean reads obtained were 40,146,590; 48,568,016; 41,588,078; 46,890,274; 43,164,170; and 44,354,944, respectively (**Table 1**). All clean reads were assembled using Trinity software, yielding a total of 80,774 unigenes with an average length of 755 nucleotides and an N50 length of 1,455 nucleotides. Functional annotation of these unigenes revealed that 38,378 were annotated in the NR database, 35,483 in KEGG, 23,617 in COG/KOG, and 28,481 in SwissProt, with 39,138 unigenes receiving at least one functional annotation (**Table 2**).

**Table 1:** Transcriptome sequencing results.

Sample	Clean data (%)
LpCK-1	40146590 (99.29%)
LpCK-2	48568016 (99.38%)
LpCK-3	41588078 (99.34%)
LpTreat-1	46890274 (99.59%)
LpTreat-2	43164170 (99.32%)
LpTreat-3	44354944 (99.33%)

**Table 2:** Characteristics of *de novo* assembled transcriptome in *L. punctata*.

Category	Statistic
Total Unigenes	80774
Average length (bp)	755
N50 length (bp)	1455
Nr	38378
KEGG	35483
COG/KOG	23617
SwissProt	28481
Annotation genes	39138

**Figure 2:**
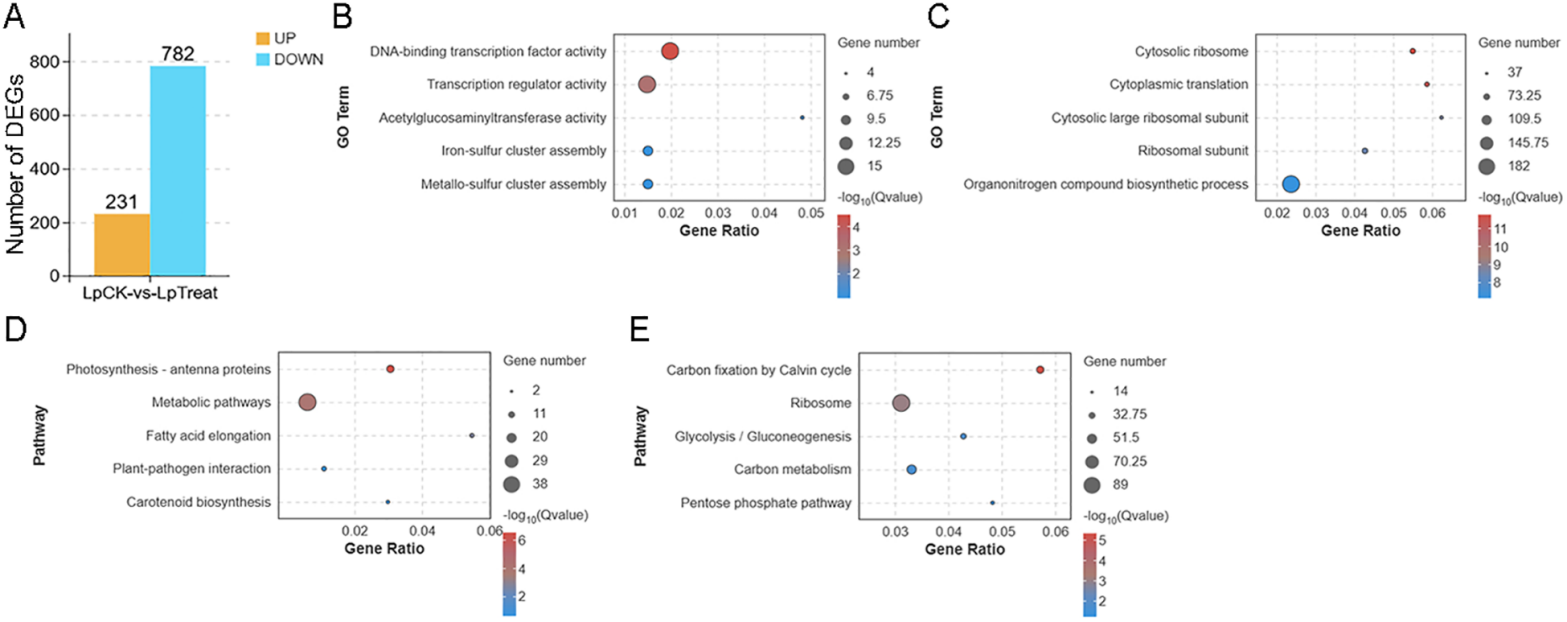
Transcriptomic analysis of *Landoltia punctata* under high nitrogen and phosphorus biogas slurry conditions. **(A)** Statistics of up- and down-regulated differentially expressed genes (DEGs). Bubble charts display GO enrichment analysis for up-regulated **(B)** and down-regulated **(C)** DEGs, and KEGG enrichment analysis for up-regulated **(D)** and down-regulated **(E)** DEGs. The top 5 significantly enriched GO terms or KEGG pathways are shown for each panel (p < 0.05).

Using |log_2_ FC| > 1 and a FDR < 0.05 as screening thresholds, a total of 1,013 DEGs were identified, including 231 significantly upregulated and 782 significantly downregulated genes (**Figure 2A**). GO functional enrichment analysis revealed that the upregulated DEGs were primarily associated with DNA-binding transcription factor activity and transcription regulator activity (**Figure 2B**), while the downregulated DEGs were significantly enriched in terms related to the Cytosolic ribosome, cytoplasmic translation, cytosolic large ribosomal subunit, and ribosomal subunit (**Figure 2C**). KEGG pathway enrichment analysis further indicated that upregulated DEGs were mainly enriched in the photosynthesis-antenna proteins pathway (**Figure 2D**), whereas downregulated DEGs were significantly enriched in pathways related to carbon fixation in photosynthetic organisms (Calvin cycle) and ribosome (**Figure 2E**).

### 3.3 DirectDIA analysis of *L. punctata*

A DirectDIA analysis was conducted on six samples: three from the blank group (LpCK-1, LpCK-2, LpCK-3) and three from the treatment group (LpTreat-1, LpTreat-2, LpTreat-3), using a peptide-level filtering threshold of FDR ≤ 0.01. This analysis yielded a total of 46,627 mass spectra, from which 44,264 peptides and 7,854 proteins were identified for subsequent differential expression analysis (**Table 3**). Functional annotation of these proteins was performed using the GO, KEGG, and KOG databases, assigning annotations to 6,482 proteins in the GO database, 3,418 in the KEGG database, and 5,540 in the KOG database. In total, 7,090 proteins were assigned at least one functional annotation across these databases, while 764 proteins remained unmatched in the aforementioned resources (**Table 3**).

**Table 3:** Characteristics of *de novo* assembled proteome in *L. punctata*.

Category	Statistic
Precursors	46627
Peptides	44264
Proteins	7854
GO	6482
KEGG	3418
KOG	5540
Annotation proteins	7090

**Figure 3:**
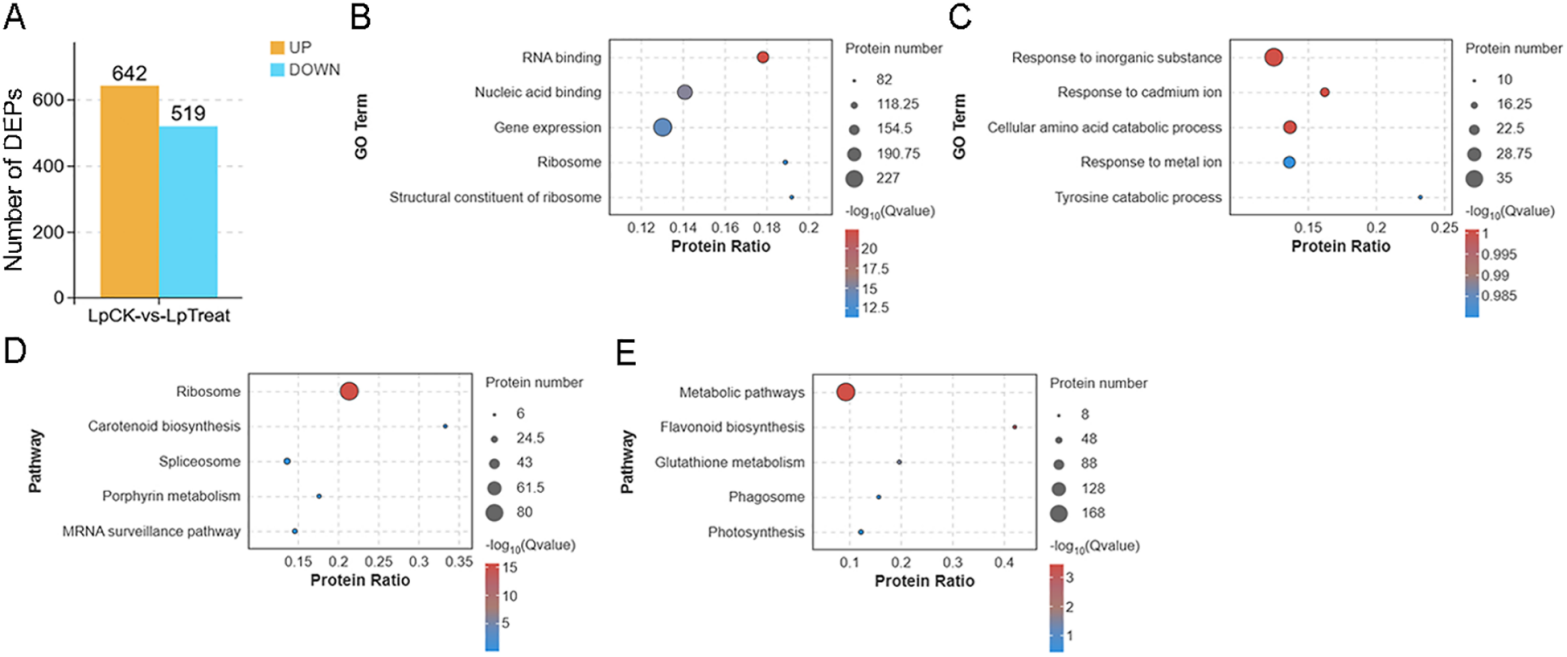
Proteomic analysis of *Landoltia punctata* under high nitrogen and phosphorus biogas slurry conditions. **(A)** Statistics of up- and down-regulated differentially expressed proteins (DEPs). Bubble charts display GO enrichment analysis of up- **(B)** and down-regulated **(C)** DEPs, and KEGG enrichment analysis of up- **(D)** and down-regulated **(E)** DEPs. The top 5 significantly enriched GO terms or KEGG pathways are shown for each panel (p < 0.05).

Based on the screening criteria of |FC| > 1.2 and p-value < 0.05, 1,161 DEPs were identified, including 642 significantly upregulated and 519 significantly downregulated proteins (**Figure 3A**). GO enrichment analysis revealed that the upregulated DEPs were mainly associated with terms including RNA binding, nucleic acid binding, gene expression, ribosome, and structural constituent of ribosome (**Figure 3B**), while the downregulated DEPs were significantly enriched in biological processes such as response to inorganic substances, response to cadmium ion, cellular amino acid catabolic process, and response to metal ions (**Figure 3C**). KEGG pathway enrichment analysis indicated that the upregulated DEPs were significantly enriched in the ribosome pathway (**Figure 3D**), whereas the downregulated DEPs were primarily enriched in metabolic pathways and flavonoid biosynthesis (**Figure 3E**).

### 3.4 Correlation analysis between mRNA and protein expression

Among the 7,854 proteins identified through directDIA analysis, corresponding mRNA expression profiles were also detected in the transcriptome. However, the overall correlation between these proteins and their corresponding mRNAs was low (R = 0.1387). In contrast, this correlation increased significantly when considering only DEPs and their corresponding mRNAs (R = 0.2362). This suggests that changes in protein and mRNA expression levels associated with the enhancement of duckweed protein content induced by biogas slurry are biologically linked.

### 3.5 Combined analysis of transcriptome and proteome of *L. punctata* treated with biogas slurry

**Figure 4:**
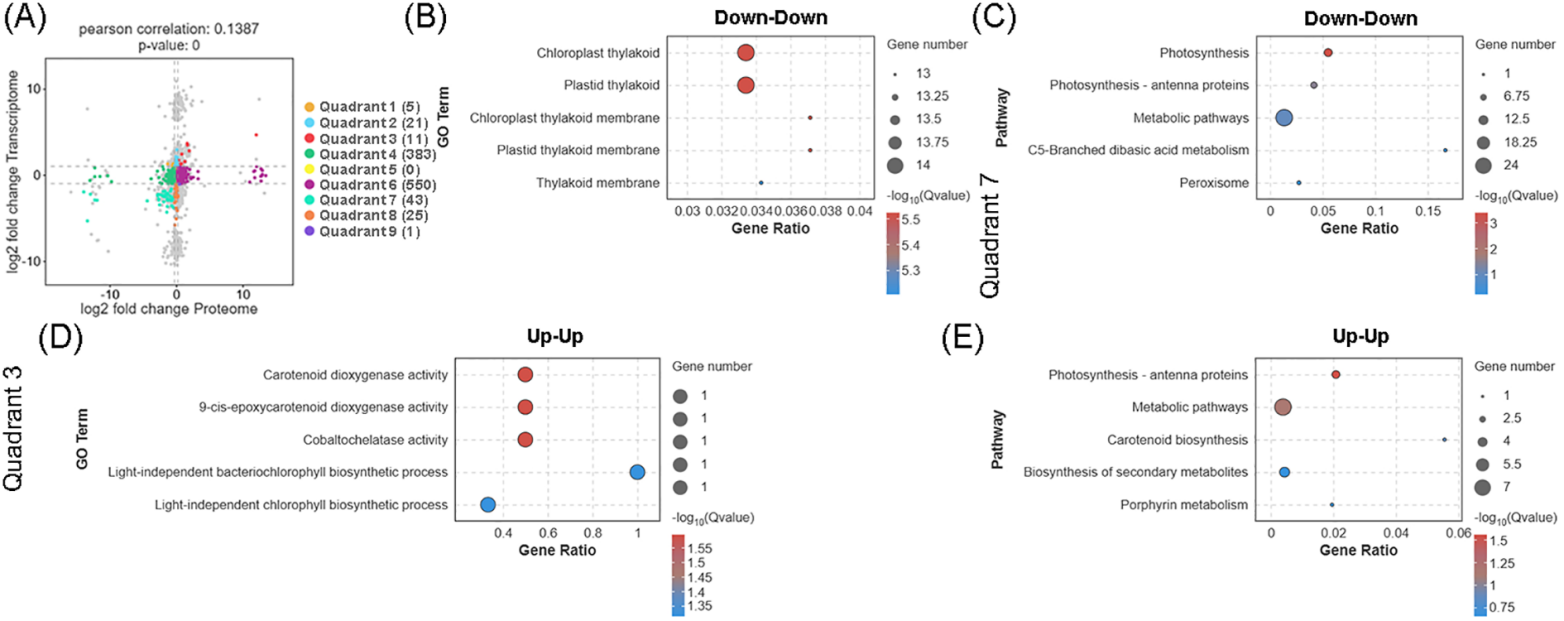
Integrated transcriptomic and proteomic analysis reveals the molecular response of *Landoltia punctata* to high nitrogen and phosphorus biogas slurry. **(A)** Nine-quadrant plot correlating differentially expressed genes (DEGs) and proteins (DEPs). Bubble charts display the GO and KEGG enrichment analyses for the 43 concordantly down-regulated **(B, C)** and 11 up-regulated **(D, E)** gene-protein pairs. “Up-Up” and “Down-Down” denote significant up- or down-regulation at both transcriptional and protein levels, respectively. The top 5 significantly enriched GO terms or KEGG pathways are shown for each panel (p < 0.05).

Overlapping analysis of DEGs and DEPs revealed that 54 genes exhibited consistent expression trends at both mRNA and protein levels in the comparison between the biogas slurry treatment group (T) and the control group (C) (**Figure 4A**), with 43 synchronously downregulated genes were enriched in chloroplast thylakoid structure and photosynthetic pathways (**Figures 4A-C**), such as *ATPF2*, which affects ATP synthase β-subunit stability, and PSBQ, a component of photosystem II (PSII). This downregulation of these genes may reduce the stability of the thylakoid membrane., impairing light energy capture and electron transport and thereby inhibiting the core light reaction processes. Conversely, 11 synchronously upregulated genes were associated with carotenoid metabolism, cofactor synthesis, and photosynthetic antenna protein pathways (**Figures 4A, D, E**). Such as *NCED1*, which catalyzes 9-cis-epoxycarotenoid cleavage to promote ABA synthesis and activate stress-responsive gene expression; CHLH, a key chlorophyll biosynthesis enzyme, whose increased expression implies a compensatory enhancement in photosynthetic capacity under stress; and LI818 (key proteins for light damage protection), whose upregulation suggests enhanced non-photochemical quenching (NPQ) to dissipate excess energy and minimize reactive oxygen species formation, while also contributing to energy balance regulation between photosystem I (PSI) and PSII. Collectively, these findings indicate that biogas slurry treatment may reshape the light energy utilization strategy of *L. punctata* by bidirectionally modulating the expression of photosynthesis-related genes.

**Figure 5:**
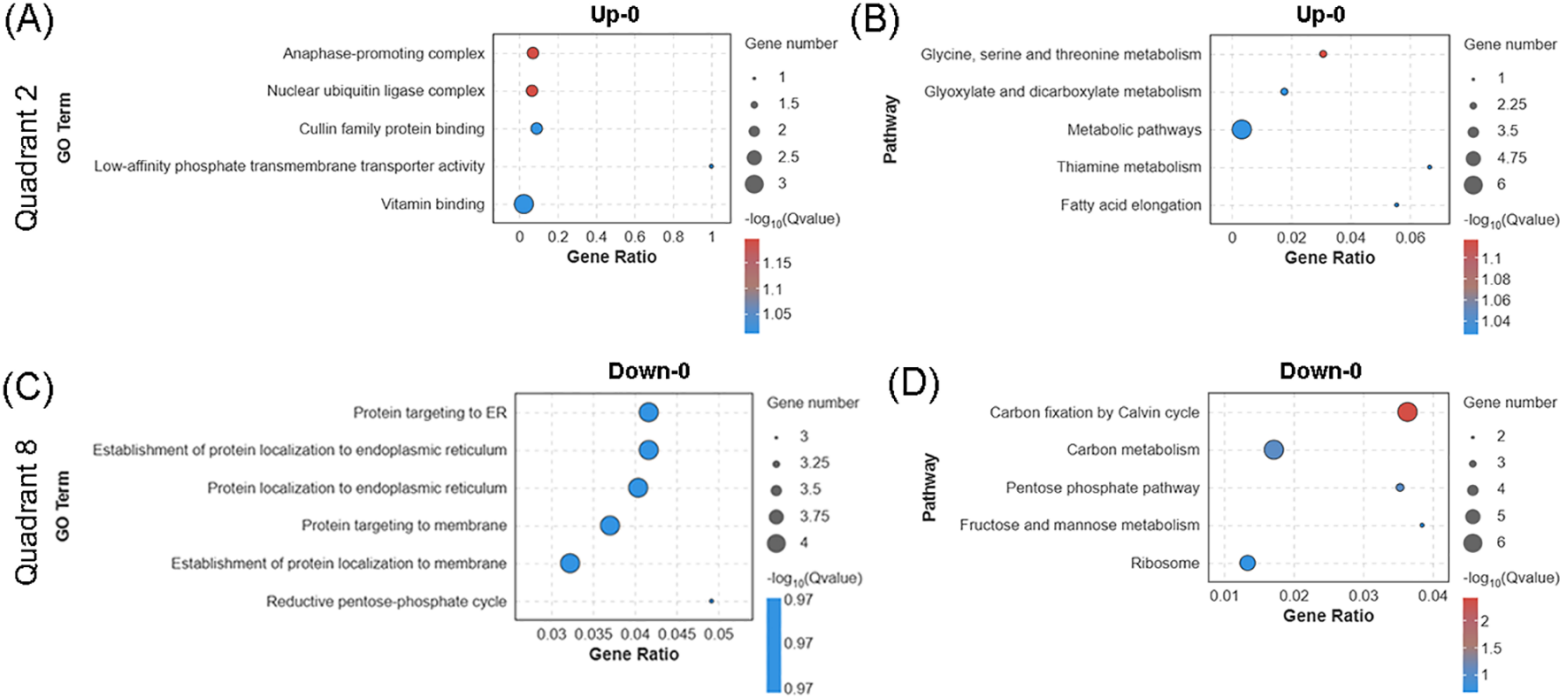
Transcript-specific regulatory responses in *Landoltia punctata* under high nitrogen and phosphorus biogas slurry. Bubble charts display GO **(A)** and KEGG **(B)** enrichment analyses for the 21 genes up-regulated solely at the transcriptional level (Up-0), as well as GO **(C)** and KEGG **(D)** enrichment for the 25 genes down-regulated only at the transcriptional level (Down-0). “Up-0” and “Down-0” denote significant up- or down-regulation solely at the transcriptional level, with no significant change at the protein level. The top 5 most significantly enriched GO terms or KEGG pathways are shown for each panel (except C) (p < 0.05).

A total of 21 upregulated and 25 downregulated DEGs did not exhibit synchronous changes at the protein level, indicating possible post-transcriptional regulation (**Figure 4A**). GO enrichment analysis showed that the upregulated DEGs were primarily involved in cell cycle regulation and vitamin metabolism (**Figure 5A**); notably, the increased transcription of *ATL6* and *PCS1*, components of the anaphase-promoting complex (APC), suggests that environmental stress affects the cell cycle progression and differentiation of duckweed. KEGG pathway analysis further indicated that these upregulated DEGs were enriched in glycine, serine and threonine metabolism and glyoxylate and dicarboxylate metabolism (**Figure 5B**), implying that biogas slurry treatment may enhance one-carbon metabolism to provide precursors for nucleic acid and amino acid synthesis. In contrast, the downregulated DEGs were enriched in pathways related to protein processing, transport, and carbon fixation by Calvin cycle (**Figure 5C, D**). Transcriptional suppression of genes involved in endoplasmic reticulum protein targeting (*RPS23*, *RPL14B*, *RPS5*, and *SEC61a*) may compromise the processing and transport of secretory proteins. Additionally, downregulation of genes involved in the reductive pentose-phosphate pathway and Calvin cycle, including *RPI3*, *GAPA*, *CSBP*, and *FBA8* (encoding fructose 1,6-bisphosphate aldolase), suggests a reduction in carbon assimilation efficiency. These results imply that biogas slurry treatment may inhibit carbon fixation through post-transcriptional mechanisms, thereby reducing photosynthetic product accumulation and redirecting carbon flow toward amino acid and protein biosynthesis pathways.

**Figure 6:**
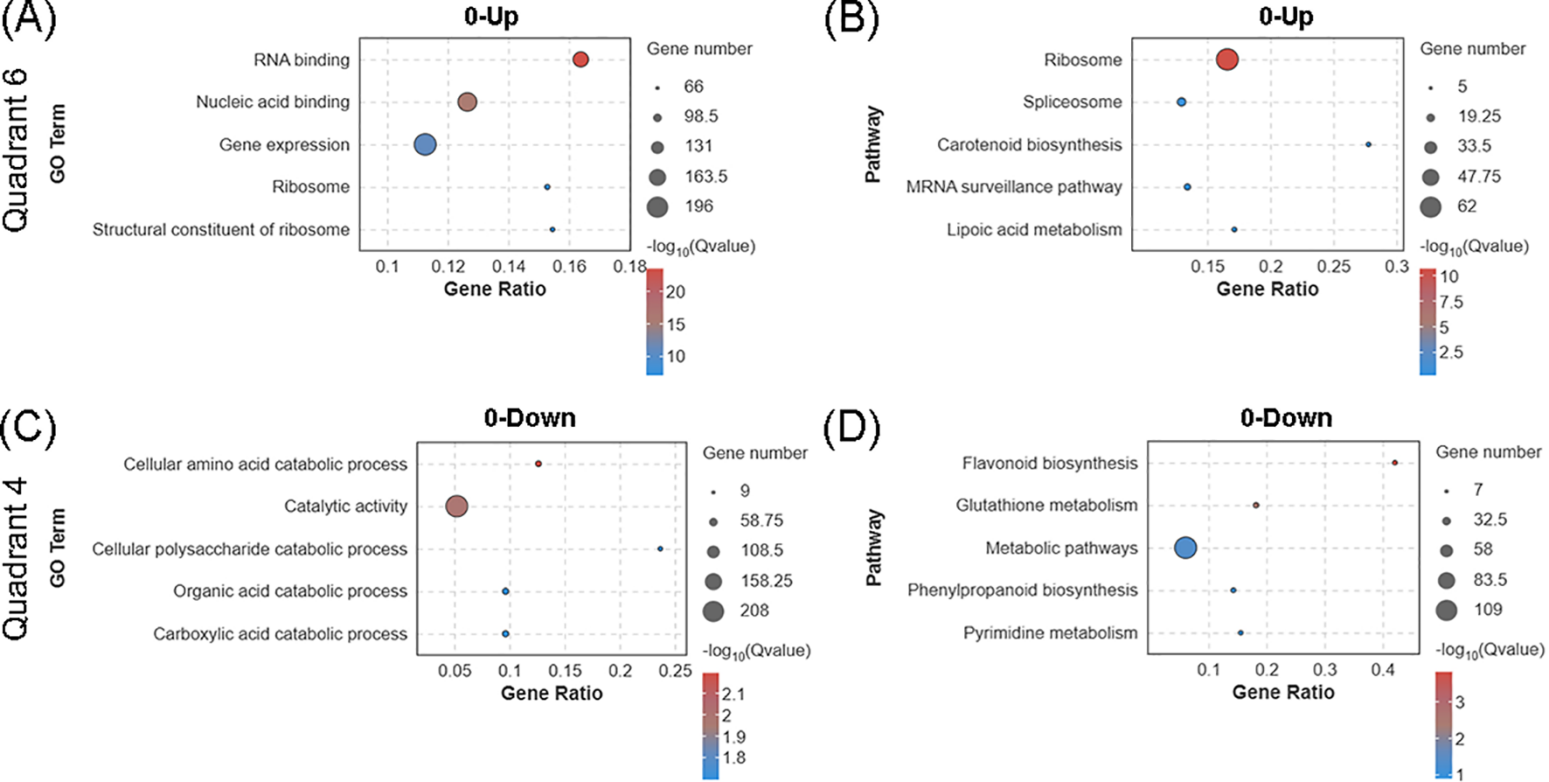
Proteomic-specific regulatory responses in *Landoltia punctata* under high nitrogen and phosphorus biogas slurry. Bubble charts display GO **(A)** and KEGG **(B)** enrichment analyses for the 550 proteins up-regulated solely at the protein level (0-Up), as well as GO **(C)** and KEGG **(D)** enrichment for the 383 proteins down-regulated only at the protein level (0-Down). “0-Up” and “0-Down” denote significant up- or down-regulation exclusively at the protein level, with no significant change at the transcriptional level. The top 5 most significantly enriched GO terms or KEGG pathways are shown for each panel (p < 0.05).

Among the DEPs exhibiting changes exclusively at the protein level, the 550 upregulated and 383 downregulated DEPs demonstrated significant functional differentiation (**Figure 4A**). GO enrichment analysis revealed that upregulated DEPs were predominantly involved in RNA binding and gene expression (**Figure 6A**); the enrichment of RNA-binding proteins (RBPs) such as SKD1, RBP47, USP106, and PHOT1 suggests an enhancement of post-transcriptional regulation, potentially modulating gene expression via increased mRNA stability or translation efficiency. KEGG pathway analysis further identified a strong enrichment in the ribosomal pathway, with 62 ribosomal subunit proteins represented (**Figure 6B**), indicating that biogas slurry treatment may accelerate protein biosynthesis by improving ribosome assembly and translation efficiency. In contrast, downregulated DEPs were enriched in pathways related to catabolism and catalytic activity (**Figure 6C, D**); for instance, downregulation of phenylalanine hydroxylase (*PAH*) and glyceraldehyde-3-phosphate dehydrogenase (*GAPDN*) in the cellular amino acid catabolic process suggests a reduction in nitrogen source degradation, potentially conserving amino acids for protein synthesis. Downregulation of key enzymes in the flavonoid biosynthesis pathway, including CCOAOMT, LAR, and F3H-2, points to an inhibition of secondary metabolite production. Additionally, changes observed in the Glutathione metabolism pathway, including suppression of GSTT1, modulation of GRC2 affecting glutathione redox balance, and decreased expression of G6PD6 and PARB (involved in the pentose phosphate pathway and the generation of reducing power), imply that the biogas slurry environment reduces the oxidative stress response in duckweed, thereby reducing its reliance on glutathione-dependent detoxification mechanisms.

In summary, the molecular regulation of *L. punctata* under biogas slurry treatment exhibits distinct hierarchical characteristics. At the level of synchronous changes in mRNA and protein expression, the inhibition of thylakoid structural protein synthesis and suppression of photosynthetic pathways appears to dynamically modulate the efficiency of light energy capture and conversion. At the mRNA level, biogas slurry treatment reconstructs carbon and nitrogen metabolic fluxes by activating genes related to the cell cycle while concurrently downregulating genes involved in carbon fixation. At the protein level, the treatment enhances RNA-binding capacity and ribosome assembly, while suppressing the expression of proteins associated with catabolism and secondary metabolism, thereby optimizing translation efficiency and reallocating energy for cellular adaptation.

### 3.6 TF-related DEGs/DEPs

**Figure 7:**
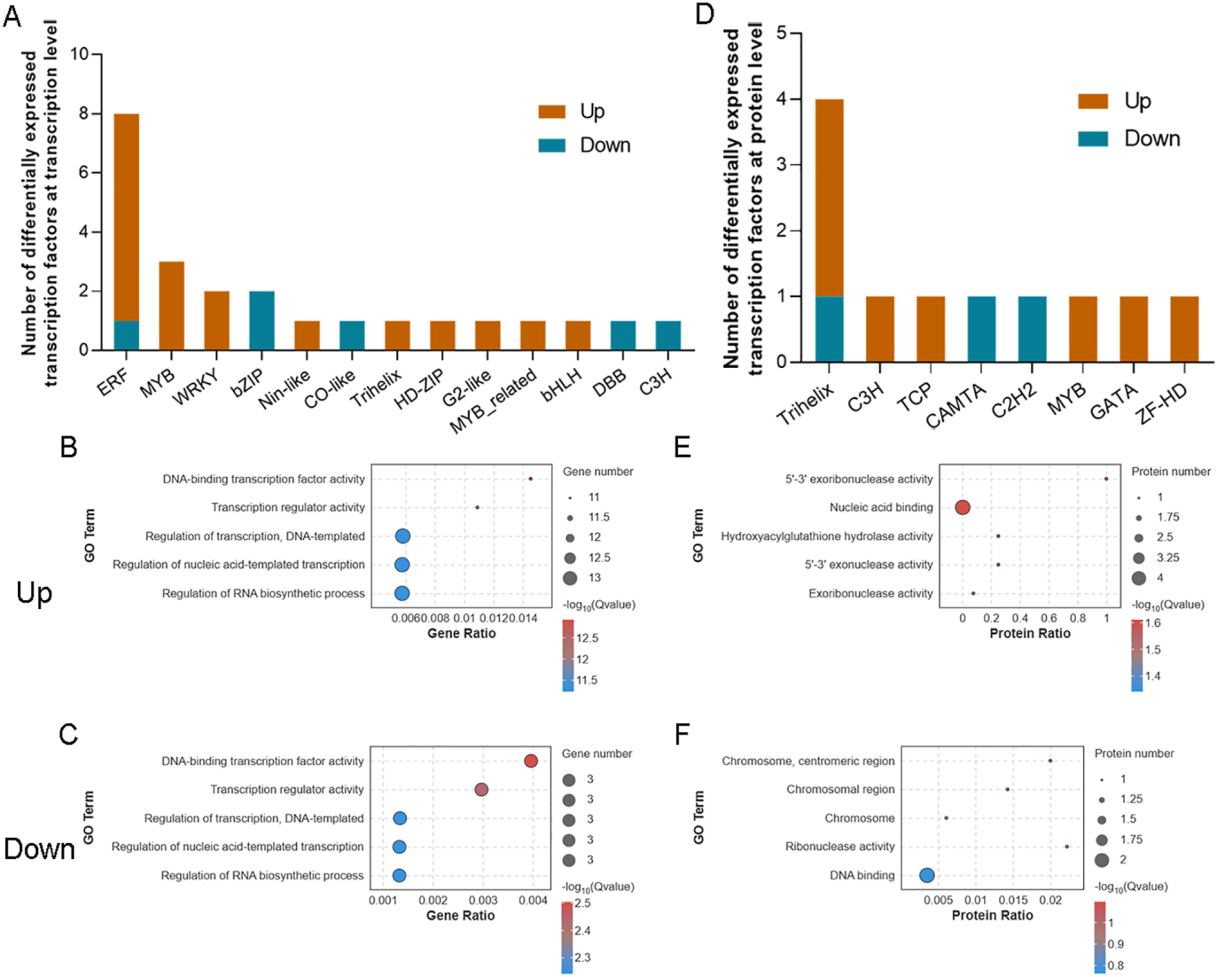
Differentially expressed transcription factors (TFs) in *Landoltia punctata* at transcriptional and protein levels under high nitrogen and phosphorus biogas slurry conditions. Number and family classification of differentially expressed TFs at the transcriptional **(A)** and protein **(D)** levels. Bubble charts display GO enrichment analysis for TFs up-regulated **(B, E)** and down-regulated **(C, F)** at the transcriptional **(B, C)** and protein **(E, F)** levels. Up- and down-regulated TFs are indicated in orange and blue, respectively. The top 5 most significantly enriched GO terms are shown for each panel (p < 0.05).

At the transcriptome level, 24 differentially expressed TF-related DEGs were identified, including 18 upregulated and 6 downregulated genes (**Figure 7A, D**). Among the upregulated DEGs, the ethylene response factor (ERF) family was the most prominent, including seven genes such as *ERF061*, *ERF014*, and *ERF8*; followed by the MYB family, with three genes such as *MYB44* and *MYB2*, and the WRKY family, represented by two genes, *WRKY11* and *WRKY57* (**Figure 7A**). These TFs are known to play vital roles in plant responses to biotic and abiotic stresses as well as in regulating growth and development. GO enrichment analysis revealed that the upregulated DEGs were significantly enriched in terms related to the TF activity. Key examples include *ERF061* and *ERF014* from the ERF family, *WRKY11* and *WRKY57* from the WRKY family, and *MYB44* from the MYB family, suggesting that biogas slurry treatment may enhance the transcriptional expression of these TFs, improve their DNA-binding capabilities, and activate downstream stress-responsive genes (**Figure 7B**). The six downregulated TF-related DEGs belonged to various families such as bZIP, CO-like, and ERF (**Figure 7C**). Notably, *bZIP44*, a part of the bZIP family, is involved in ABA signaling and stress response; its downregulation may reduce cellular sensitivity to osmotic stress. Similarly, *COL4*, a member of the CO-like family associated with photoperiod regulation, may impact the photosynthetic rhythm of *L. punctata* due to its reduced expression.

At the proteomic level, eleven differentially expressed TF-related DEPs were identified, including eight upregulated and three downregulated DEPs (**Figure 7D**). The upregulated DEPs comprised members of the Trihelix family (RNJ and DF1), C3H family (Os02g0194200), TCP family (TCP9), MYB family (CDC5), GATA family (GATA17), and ZF-HD family (ZHD1) (**Figure 7D**). Trihelix TFs are known to play roles in plant growth, development, and responses to abiotic stress, while the upregulation of C3H family proteins may be associated with RNA processing and transcriptional regulation. Similarly, upregulated TCP TFs could influence cell growth and differentiation, MYB proteins might contribute to cell cycle regulation, and GATA family members are implicated in the regulation of the carbon/nitrogen balance in plants. GO enrichment analysis revealed that several upregulated DEPs, including RNJ, Os02g0194200, GATA17, and ZHD1, were enriched in nucleic acid binding functions (**Figure 7E**), suggesting that these TFs may regulate downstream gene expression and impact cellular metabolic pathways and signal transduction at the protein level. Among the downregulated DEPs were TFs from the Trihelix, CAMTA, and C2H2 families (**Figure 7F**). CAMTA TFs are involved in plant immune responses and calcium signaling, whereas C2H2 zinc finger proteins serve as key regulators of plant development and stress responses. Notably, no TFs exhibited consistent expression patterns at both the mRNA and protein levels, indicating that TFs in duckweed under the high nitrogen and phosphorus environmental stress induced by biogas slurry, may be subject to complex post-transcriptional regulatory mechanisms.

### 3.7 Network analysis of protein-protein interaction

**Figure 8:**
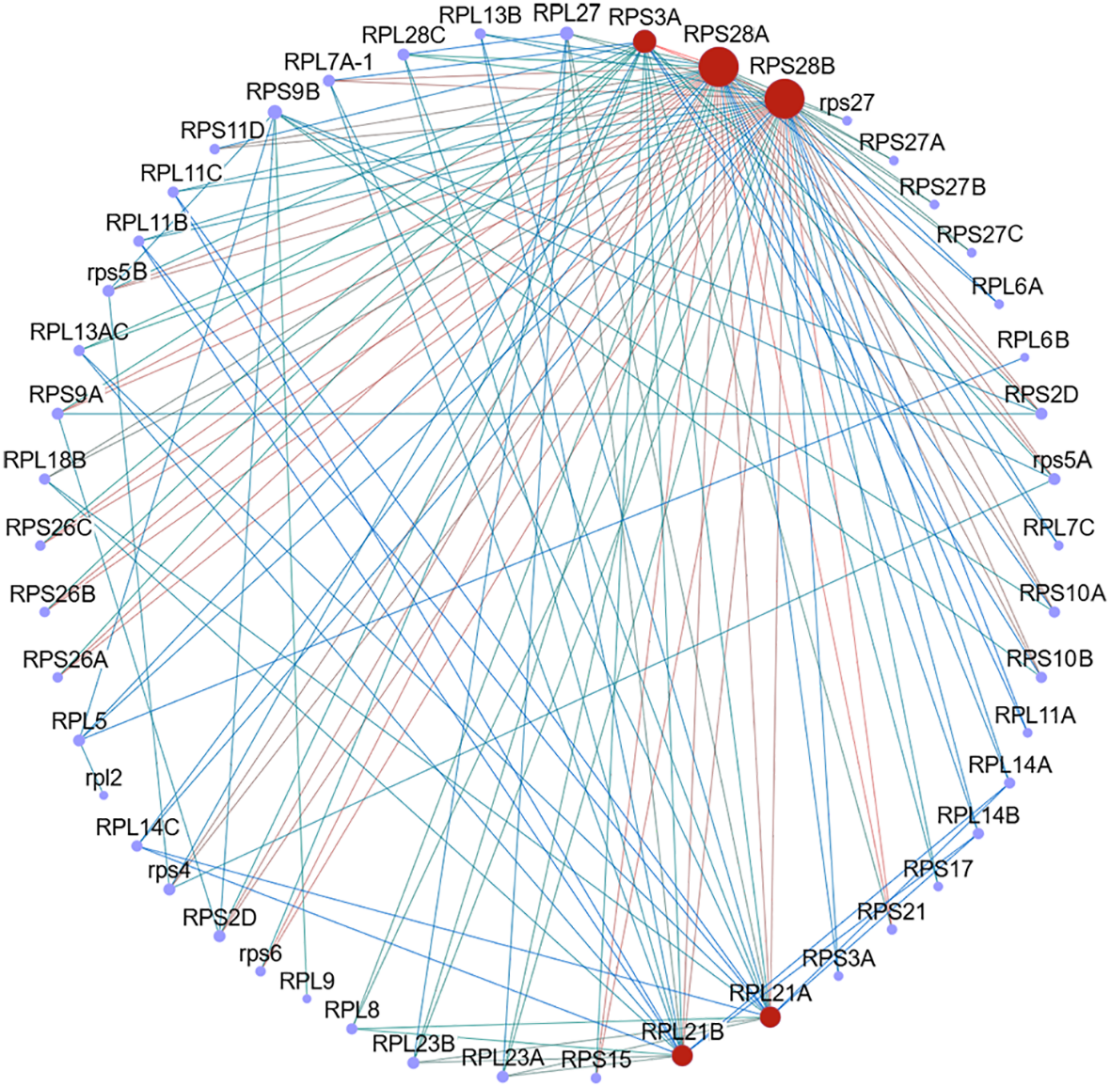
Protein-protein interaction (PPI) network analysis of differentially expressed proteins (DEPs). Red nodes represent the top 5 hub genes identified within the enriched PPI clusters.

Ribosomes are essential sites of protein synthesis, and to investigate the key proteins responsible for the increase in protein content in duckweed following biogas slurry treatment, as well as to understand their interactions and functional roles, this study identified 62 DEPs that were upregulated in the proteome and enriched in the ribosome pathway, based on KEGG analysis. To further explore protein interactions, a target PPI network was constructed using interaction data from the STRING database (http://string-db.org), with interactions filtered by a combined score ≥ 980. The resulting PPI network revealed five key hub proteins, *RPS28A*, *RPS28B*, *RPS3A*, *RPL21A1*, and *RPL21A2* (**Figure 8**), which showed putative interactions with 49, 49, 42, 39, and 39 of the 62 DEPs, respectively. These findings suggest that these ribosomal proteins (RPs) may play a central role in regulating protein synthesis and contribute significantly to the observed increase in protein content in duckweed under biogas slurry treatment.

### 3.8 qPCR verification

**Figure 9:**
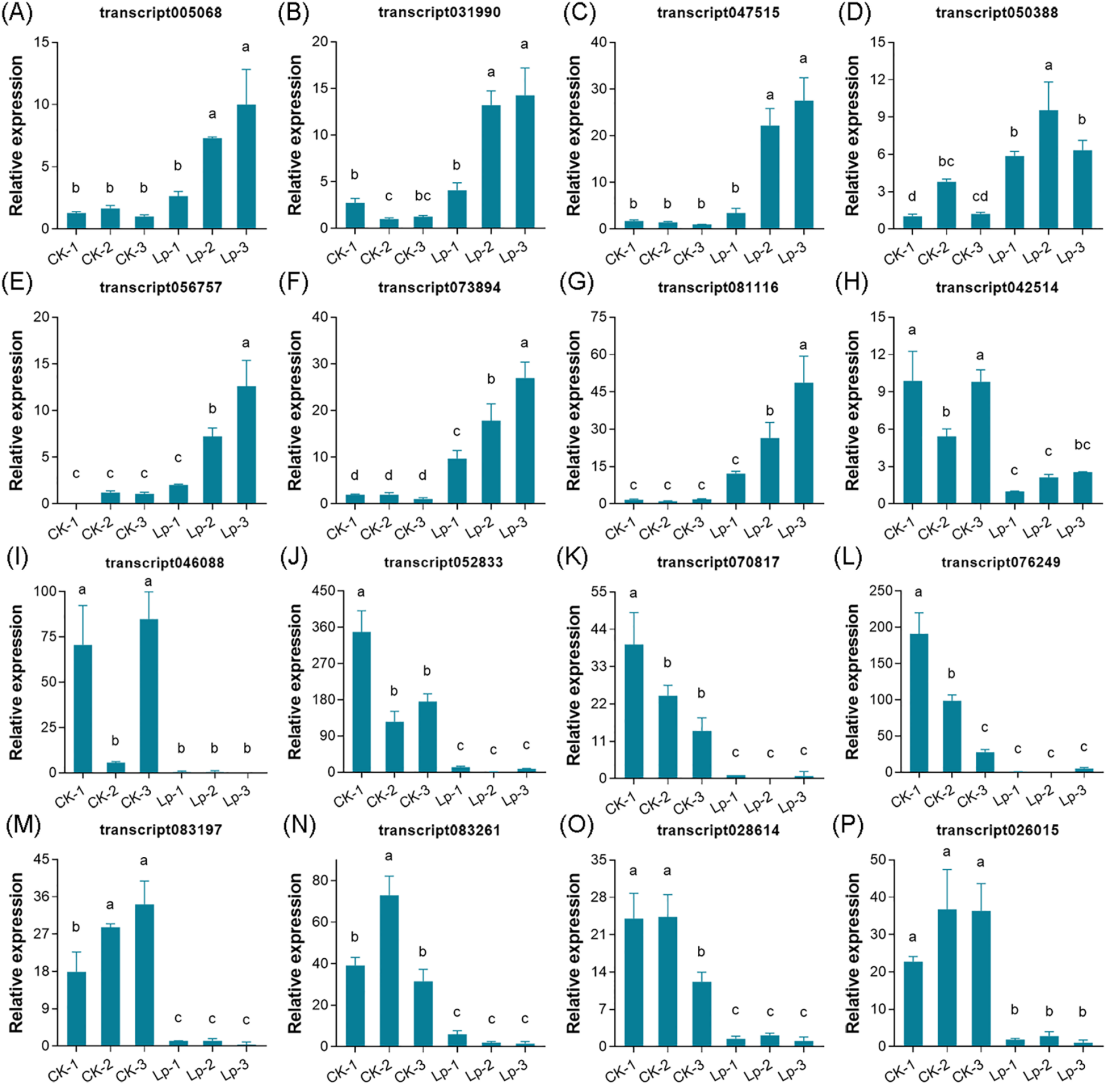
Gene expression levels detected by qRT-PCR. **(A-P)** Expression levels of transcripts transcript005068, transcript031990, transcript047515, transcript050388, transcript056757, transcript073894, transcript081116, transcript042514, transcript046088, transcript052833, transcript070817, transcript076249, transcript083197, transcript083261, transcript028614, and transcript026015 in the control and biogas slurry treatment groups, respectively. Data are presented as mean ± standard deviation of three biological replicates. Different letters above bars indicate significant differences according to Tukey’s HSD test (P < 0.05).

To validate the reliability of the transcriptome sequencing data, 16 genes, including seven upregulated and nine downregulated genes, were selected for qRT-PCR analysis. The qRT-PCR results demonstrated that the expression trends of all selected genes were consistent with those observed in the transcriptome data (**Figure 9**), thereby confirming the accuracy and reliability of the transcriptome sequencing results.

## 4 Discussion

### 4.1 The enhancing effect of biogas slurry on the protein content of *L. punctata*

Pig farm biogas slurry is rich in essential nutrients beneficial for plant growth (Zhan et al., 2020; Sánchez-Zurano et al., 2021). In agricultural practice, several applications of pig farm biogas slurry have been documented. For example, using biogas slurry to irrigate watermelons has been shown to reduce chemical fertilizers requirements without significantly impacting yield (Lu et al., 2010). Additionally, the combined application of biogas slurry and biochar has been found to enhance the protein, soluble sugar, and starch content in lotus root (Zhang et al., 2024a).

The purification of sewage using duckweed has become a prominent research focus in recent years. Studies have demonstrated that duckweed can effectively remove key nutrients such as nitrogen and phosphorus from aquatic environments, while also exhibiting a strong adsorption capacity for heavy metals like cadmium and arsenic (Liu et al., 2020; Iyer et al., 2024; Li et al., 2025). Preliminary research conducted by our team further supports these findings, showing that duckweed achieved removal rates of 54.69% for chemical oxygen demand (COD), 86.89% for total nitrogen (TN), 97.25% for ammonia nitrogen (NH_3_-N), and 85.22% for total phosphorus (TP) in wastewater (Li et al., 2025), highlighting its significant potential for practical application in wastewater treatment.

Although biogas slurry is known to be rich in nutrients, there have been no prior reports on whether its use in cultivating duckweed can enhance the plants’ protein content. This study found that supplementing the basic culture medium with 1.5%, 2%, 3%, 4%, and 5% biogas slurry increased the protein content of duckweed to varying extents. Notably, at a concentration of 4%, the protein content was significantly higher than that of the control group. However, when the concentration exceeded 4%, a decline in protein content was observed, which may be attributed to elevated levels of chlorine-containing salts in the higher-concentration biogas slurry surpassing duckweed’s tolerance threshold, thereby inhibiting its growth (Scott et al., 2023; Van Meter and Ceisel, 2024). These findings not only provide a theoretical basis for the sustainable cultivation of high-protein duckweed but also facilitate the exploration of molecular mechanisms underlying protein content enhancement induced by biogas slurry, through the application of high-throughput sequencing technologies. This lays a strong foundation for future molecular breeding research.

### 4.2 Low overlap of transcriptome proteome DEGs/DEPs indicates that post transcriptional regulation dominates biogas slurry to induce duckweed protein elevation

Post-transcriptional regulation refers to the control of gene expression through various mechanisms that occur after mRNA transcription, including mRNA splicing, editing, transport, translation, and degradation, as well as protein modification, localization, and degradation (Wang et al., 2020). This regulatory process plays a vital role in plant growth and development (Romanowski and Yanovsky, 2015), responses to environmental stresses (Tong et al., 2020), metabolic regulation (Cheng et al., 2023), and the modulation of immune responses (Gong et al., 2022).

This study identified 1,013 DEGs and 1,161 DEPs at the transcriptome and proteome levels, respectively. KEGG enrichment analysis revealed that the upregulated and downregulated DEGs and DEPs did not accumulate in the same pathways. Joint analysis further showed that only 54 genes exhibited synchronous differential expression, either upregulation or downregulation, at both the mRNA and protein levels. Moreover, correlation analysis between mRNA and protein expression yielded a low correlation coefficient (R = 0.1387), indicating a poor correspondence between transcriptomic and proteomic changes. Notably, while downregulated DEGs were significantly enriched in the ribosome pathway, this same pathway showed significant enrichment among upregulated DEPs; yet no genes within this pathway displayed consistent trends across both levels. This apparent contradiction strongly suggests that post-transcriptional regulation plays a dominant role in the protein content increase in duckweed induced by biogas slurry treatment. Supporting this conclusion, a previous study by Fu et al (Fu et al., 2024). integrated transcriptomic and proteomic data to investigate the molecular mechanism by which SA induces flowering in *L. gibba*. Their findings revealed that changes in mRNA levels were not aligned with corresponding protein abundance, highlighting the critical and extensive role of post-transcriptional mechanisms in the SA-mediated flowering signaling pathway. SA was shown to finely regulate the synthesis, function, and stability of flowering-related proteins through non-transcription-dependent pathways, ultimately driving the flowering process. The present study reaffirms the significance and universality of post-transcriptional regulation in duckweed’s response to abiotic stress.

### 4.3 Regulation of expression of ribosome-related genes

Under abiotic stress, gene expression is regulated not only at the transcriptional level but also through post-transcriptional mechanisms, including mRNA processing and modification, regulation of mRNA stability, and translation efficiency control (Floris et al., 2009). Ribosomes, as the central machinery for protein synthesis, consist of approximately 81 RPs and four types of rRNA in plants, facilitating the translation of genetic information into functional proteins (Xiong et al., 2021; Dias-Fields and Adamala, 2022). Therefore, understanding the regulation of ribosome-related genes at both transcriptional and post-transcriptional levels is crucial for enhancing protein content in duckweed. In this study, the expression patterns of ribosome-related genes revealed a low correlation, and even opposing trends, between the transcriptome and proteome: 89 downregulated DEGs were enriched in the ribosome pathway at the transcriptomic level, while 80 upregulated DEPs were enriched in the same pathway at the proteomic level. Joint analysis further identified 148 and 62 DEPs, exhibiting no significant transcriptional changes but significant protein-level upregulation, enriched in RNA-binding and ribosome-related pathways, respectively. These results suggest that ribosome-related genes undergo post-transcriptional regulation in response to biogas slurry-induced environmental stress. Previous studies have shown that under abiotic stress conditions such as salinity, drought, or temperature fluctuations, ribosomes can undergo structural changes, referred to as ribosome heterogeneity, enabling selective translation of specific mRNAs to enhance plant adaptability (Moin et al., 2016; Dias-Fields and Adamala, 2022). RBPs, as key mediators of post-transcriptional regulation, influence mRNA processing, transport, stability, and translation efficiency by interacting directly with mRNA (Rehman et al., 2024). Notably, the pig farm biogas slurry used in this study exhibited high nitrogen and phosphorus loads, with TN and TP contents of 980 mg/L and 90 mg/L, respectively. Based on these findings, it is speculated that under such high-nutrient stress conditions, duckweed may preferentially translate mRNAs of ribosome-related genes via upregulated RBPs, thereby ensuring the continued synthesis of ribosomal assembly proteins and maintaining high protein synthesis efficiency, even when transcriptional activity is downregulated or unchanged. Additionally, key enzymes involved in amino acid catabolism, such as PAH and GAPDN, exhibited decreased expression at the protein level, suggesting that amino acid degradation processes were suppressed, thereby conserving essential substrates for protein biosynthesis.

*RPS28A*, *RPS28B*, *RPS3A*, *RPL21A1*, and *RPL21A2* were identified as the top five hub genes in the PPI analysis, highlighting their central role in the enhancement of protein content in *L. punctata* induced by biogas slurry. *RPS28* is a core component of the 40S ribosomal subunit, directly interacting with the 5’ untranslated region of mRNA and playing a crucial role in ribosome biogenesis by influencing the formation of translation initiation complexes (Jiao et al., 2021). In Arabidopsis, mutations in *RPS28B* have been shown to disrupt rRNA processing, impair ribosome maturation, and result in stunted plant growth (Horiguchi et al., 2011), emphasizing its essential function in protein synthesis and developmental regulation. As a key component of the 40S ribosomal subunit, *RPS3A* cooperates with *RPS19* and *RPS13* to form binding sites for translation initiation factors eIF-2 and eIF-3, directly affecting ribosome stability and function (Uzair et al., 2021). In rice, its absence disrupts 40S-eIF-3 binding, inhibiting synthesis of regulatory proteins like ARFs and OsWOX3A (Uzair et al., 2021). *RPS3A* also contributes to selective mRNA translation via ribosome heterogeneity. In mulberry, altered *RPS3A* expression affects stress-related protein synthesis, and its loss causes ribosome disintegration, protein synthesis suppression, and cell apoptosis (Qian et al., 2016).

Both *RPL21A1* and *RPL21A2* belong to the RPL21e family and serve as essential components of the large ribosomal subunit (60S). In archaea, *RPL21e*, and in bacteria, its functional analog *RPL27*, are located in key structural regions of the ribosomal subunit, suggesting that these proteins may play a central role in ribosome assembly and overall function (Sun et al., 2017). Their conserved positioning across domains of life highlights their importance in maintaining the structural integrity and translational efficiency of ribosomes.

In this study, the transcriptional levels of *RPS28A*, *RPS28B*, *RPS3A*, *RPL21A1*, and *RPL21A2* showed no significant changes; however, their protein levels were significantly upregulated, suggesting that these genes contribute to enhanced protein synthesis in *L. punctata* through post-transcriptional regulation. This finding underscores the importance of translational control mechanisms in modulating RP abundance and highlights the role of post-transcriptional processes in the biogas slurry-induced increase in protein content.

### 4.4 Expression regulation of photosynthesis-related genes

Photosynthesis, occurring in chloroplasts, converts light energy into chemical energy through light and dark reactions. The light reaction splits water to release oxygen and produces ATP and NADPH, which drive the dark reaction, where CO_2_ is fixed into carbohydrates like glucose (Hossain et al., 2022). Antenna proteins aid by capturing light and transferring energy to photosystems II and I, activating the electron transport chain and generating ATP and NADPH (Di et al., 2023). These molecules support the Calvin cycle and contribute to protein synthesis (Zhen et al., 2017; Liu et al., 2022), as carbon intermediates like 3-phosphoglycerate enter amino acid biosynthesis (Zhen et al., 2017). In this study, transcriptomic analysis showed upregulation of genes in the antenna protein pathway and downregulation in the Calvin cycle. Combined transcriptome–proteome analysis revealed 54 DEGs with consistent trends; 11 (e.g., *CAB91R*, *LHCB43*, *LI818*) were co-upregulated in the antenna protein pathway, while 43, including four antenna protein genes (*LHCA4*, *LHCA5*, *LHCB4*, *LHCB5*), were co-downregulated in the photosynthetic core and thylakoid-related pathways. These findings suggest that under the high nitrogen and phosphorus stress imposed by biogas slurry, the photosynthetic system of duckweed was reprogrammed for environmental adaptation. The dual-omics downregulation of key antenna proteins (such as LHCA4/5 and LHCB4/5) and thylakoid structure-related genes, along with the suppressed expression of Calvin cycle genes such as *RBCS2*, *PGK*, *PRKA*, and *GapC*, encoding RuBisCO, PGK, PRK, and GAPDH, respectively, indicates that duckweed actively reduced its light-harvesting capacity and carbon fixation, likely reallocating the conserved carbon skeletons and energy (ATP/NADPH) toward nitrogen assimilation and amino acid biosynthesis. Furthermore, the LI818 protein, a crucial component of rapidly inducible non-photochemical quenching (NPQ), plays an important role in light adaptation and photoprotection in plants (Sturm et al., 2013; Lokstein et al., 2021). The dual omics upregulation of *LI818* with specific photosynthetic antenna protein genes such as *LHCB43* and *CAB91R* suggests that duckweed may have strengthened photoprotective mechanisms such as NPQ under strong light culture conditions (6000 LUX) stress. While dissipating excess light energy in the form of thermal energy, it also maintains a certain basic light capture efficiency to maintain cellular basic energy supply. Notably, the increased transcriptional activity of photosynthetic antenna protein genes was not matched by corresponding upregulation at the protein level, mirroring observations from the ribosomal pathway and suggesting that photosynthetic genes are also subject to substantial post-transcriptional regulation under biogas slurry-induced stress.

## 5 Conclusion

**Figure 10:**
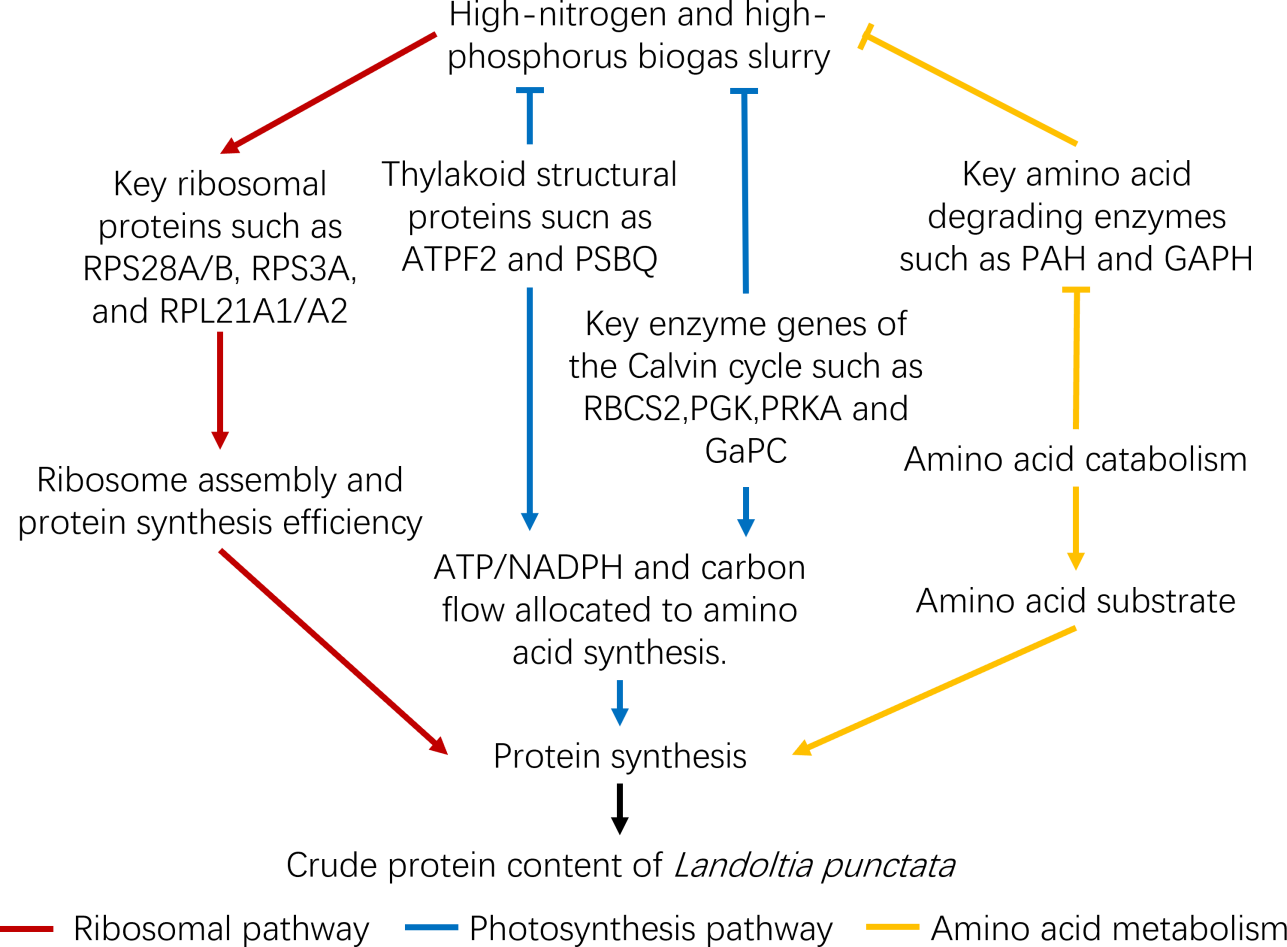
Proposed molecular regulatory model for increased crude protein content in *Landoltia punctata* induced by high nitrogen and phosphorus biogas slurry. Arrows and T-bar symbols represent positive and negative (inhibitory) regulatory relationships, respectively. Regulatory relationships involved in the ribosomal pathway, amino acid metabolism pathways, and photosynthetic phase are indicated in red, orange, and blue, respectively.

This study investigates the impact of high nitrogen and phosphorus environmental stress from biogas slurry on protein accumulation in *L. punctata* and explores its molecular regulatory mechanisms through integrated transcriptomic and proteomic analyses. The results demonstrated that supplementing the basal culture medium with 1.5%-5% biogas slurry significantly increased protein content in duckweed, with 4% identified as the optimal concentration. Beyond this threshold, protein content declined due to chloride-induced stress associated with higher biogas slurry concentrations, thereby inhibiting plant growth. Molecular analysis revealed that the high nitrogen and phosphorus stress enhanced protein synthesis efficiency through post-transcriptional regulation of ribosomal and photosynthesis-related pathways (**Figure 10**). Specifically, an expression pattern characterized by “transcriptionally invariant but translationally upregulated” was observed in ribosomal pathway components, promoting ribosome assembly and translation efficiency, while simultaneously reducing intracellular amino acid degradation, thereby enhancing protein synthesis at the post-transcriptional level. Additionally, adaptive reprogramming of the photosynthetic system was observed, where duckweed strategically downregulated light energy capture and Calvin cycle activity, redirecting ATP, NADPH, and carbon skeleton intermediates from photosynthetic light reactions toward nitrogen assimilation and amino acid biosynthesis. This metabolic shift provided both the energy and precursors required for increased protein accumulation. Importantly, this study is the first to reveal the pivotal role of post-transcriptional regulation in duckweed’s response to high nitrogen and phosphorus stress in biogas slurry, offering valuable insights and a theoretical foundation for molecular breeding strategies targeting RPS/RPL gene families to develop high-protein duckweed cultivars.

## Data Availability

The mass spectrometry proteomics data have been deposited to the ProteomeXchange Consortium (https://proteomecentral.proteomexchange.org) via the iProX partner repository with the dataset identifier PXD068264. The raw RNA-seq data have been deposited in the NCBI Sequence Read Archive (SRA) (https://www.ncbi.nlm.nih.gov/sra) under the accession number PRJNA1311761.
