# Supplementary material for: Post-transcriptional regulation dominates protein biosynthesis in Landoltia punctata under biogas slurry stress
Source: Front Plant Sci. 2025 Nov 20;16:1694864. doi: 10.3389/fpls.2025.1694864 (PMC12676286; doi:10.3389/fpls.2025.1694864)
Supplement: Supplementary file 1 [file Table1.docx]

**Table S1.** Oligonucleotide primers used in this study.

| Primer name | Primer Sequences（5’-3’） |
| --- | --- |
| Lp-actin-F | TGAACCCTAAAGCCAACAGA |
| Lp-actin-R | CCGTGGTGGTGAAAGAGTAA |
| 081116-F | CCAACCCGGCCCCATCATGT |
| 081116-R | GGTCGTAGTTCTTCGTGTCGTC |
| 056757-F | TATGCGGAGTCCTGCTGTGAA |
| 056757-R | GCTGCGTCTGTCTTGGTTGC |
| 047515-F | CAGCATTGACCAGGGCGTAA |
| 047515-R | TGAGATGGTTGACCACGAAGG |
| 073894-F | AAGTTTTCGCCCCGTAT |
| 073894-R | AACACCCACAACCAGACAC |
| 005068-F | AGCATCCCATCAACAAGCG |
| 005068-R | CCCTGAGTCTGGACGACATTAG |
| 050388-F | TCGTCCTTGCCTTTACCTT |
| 050388-R | CGAATCGGATGAACTGATGT |
| 031990-F | GGGCGGGTATGAGATGGA |
| 031990-R | TGTCGTGGTGCGATTGG |
| 042514-F | CGCCGCCCAGAAAGACTACAC |
| 042514-R | GGCATCGGAAGGAGCAAAGA |
| 076249-F | TCATCCGCAATCCACGACTA |
| 076249-R | CCTCCTTCAGCCTGTCACCC |
| 046088-F | ATTGAGCGGCACTCCTGGTA |
| 046088-R | ATTGAAGGTTGGCTTGATGG |
| 052833-F | TTCAATACGCCAACCCA |
| 052833-R | TAAAACGAAGCCATCTCAT |
| 083197-F | TGTGGAGGGCGGGTAAG |
| 083197-R | CGAGCAGGACAGAGCAAAT |
| 070817-F | CGGTCGCCATTTCTGTG |
| 070817-R | CTCCCTTATTGACTGCTTTGT |
| 083261-F | GCAGTTCAGTCGGCTATCA |
| 083261-R | CCAGCACAATCCCAGTCA |
| 028614-F | TGTCTCCTGAGGGAATGTGT |
| 028614-R | GCCAGCAAACAGGGAACT |
| 026015-F | CGGTGCGGGTTGTTGAT |
| 026015-R | GAGGAGTCGCCGTGCTT |
